# *Sutterella* and its metabolic pathways positively correlate with vaccine-elicited antibody responses in infant rhesus macaques

**DOI:** 10.3389/fimmu.2023.1283343

**Published:** 2023-12-06

**Authors:** Danting Jiang, Ria Goswami, Maria Dennis, Holly Heimsath, Pamela A. Kozlowski, Amir Ardeshir, Koen K. A. Van Rompay, Kristina De Paris, Sallie R. Permar, Neeraj K. Surana

**Affiliations:** ^1^Department of Pediatrics, Duke University School of Medicine, Durham, NC, United States; ^2^Program in Computational Biology and Bioinformatics, Duke University School of Medicine, Durham, NC, United States; ^3^Department of Pediatrics, Weill Cornell Medicine, New York, NY, United States; ^4^Duke Human Vaccine Institute, Duke University School of Medicine, Durham, NC, United States; ^5^Department of Microbiology, Immunology and Parasitology, Louisiana State University Health Sciences Center, New Orleans, LA, United States; ^6^California National Primate Research Center, University of California, Davis, Davis, CA, United States; ^7^Department of Microbiology and Immunology, University of North Carolina, Chapel Hill, NC, United States; ^8^Department of Molecular Genetics and Microbiology, Duke University School of Medicine, Durham, NC, United States; ^9^Department of Integrative Immunobiology, Duke University School of Medicine, Durham, NC, United States; ^10^Department of Cell Biology, Duke University School of Medicine, Durham, NC, United States

**Keywords:** gut microbiota, vaccine responses, infants, macaque, *Sutterella*, metabolites

## Abstract

**Introduction:**

It is becoming clearer that the microbiota helps drive responses to vaccines; however, little is known about the underlying mechanism. In this study, we aimed to identify microbial features that are associated with vaccine immunogenicity in infant rhesus macaques.

**Methods:**

We analyzed 16S rRNA gene sequencing data of 215 fecal samples collected at multiple timepoints from 64 nursery-reared infant macaques that received various HIV vaccine regimens. PERMANOVA tests were performed to determine factors affecting composition of the gut microbiota throughout the first eight months of life in these monkeys. We used DESeq2 to identify differentially abundant bacterial taxa, PICRUSt2 to impute metagenomic information, and mass spectrophotometry to determine levels of fecal short-chain fatty acids and bile acids.

**Results:**

Composition of the early-life gut microbial communities in nursery-reared rhesus macaques from the same animal care facility was driven by age, birth year, and vaccination status. We identified a *Sutterella* and a *Rodentibacter* species that positively correlated with vaccine-elicited antibody responses, with the *Sutterella* species exhibiting more robust findings. Analysis of *Sutterella*-related metagenomic data revealed five metabolic pathways that significantly correlated with improved antibody responses following HIV vaccination. Given these pathways have been associated with short-chain fatty acids and bile acids, we quantified the fecal concentration of these metabolites and found several that correlated with higher levels of HIV immunogen-elicited plasma IgG.

**Discussion:**

Our findings highlight an intricate bidirectional relationship between the microbiota and vaccines, where multiple aspects of the vaccination regimen modulate the microbiota and specific microbial features facilitate vaccine responses. An improved understanding of this microbiota–vaccine interplay will help develop more effective vaccines, particularly those that are tailored for early life.

## 1 Introduction

Second only to the provision of clean water, vaccinations have been the most effective public health intervention in the prevention of serious infectious diseases (1), with the global implementation of immunization programs providing a cost-effective platform to reduce millions of infection-related deaths each year (2). The effects of vaccines are mediated by antigen-specific antibodies and, in some cases, effector T cell responses. Although vaccines are clearly effective on a population scale, the magnitude of the immune response to vaccines can vary among individuals by 10–100-fold (3–5), which leads to inadequate responses in some populations (6).

While many factors (e.g., genetics, maternal antibody levels, prior antigen exposures) can affect vaccine immunogenicity, there is increasing recognition that the microbiota—the vast collection of microbes that colonize the entire body—may also impact responses to vaccines given its integral role in modulating immune responses and human health (6–9). Numerous clinical studies have associated variability in vaccine responses with specific differences in the microbiome (6, 10–12), and these correlations have been partially confirmed in animal studies that demonstrate the microbiota regulates responses to non-adjuvanted viral subunit vaccines, with less clear effect on live or adjuvanted protein vaccines (13, 14). Moreover, an interventional clinical trial revealed that depletion of the microbiota with broad-spectrum antibiotics impaired neutralizing and antigen-binding antibody responses following influenza vaccination only in those individuals who had low baseline antibody titers, with no effect in those with pre-existing immunity (15). As such, the microbiota may serve as a natural adjuvant (16–19), enhancing primary responses to vaccines of specific types. However, there is little known regarding the specific microbial features that positively impact vaccine responses.

In addition, the age at which one is vaccinated may also contribute to variability in responses. Infants worldwide are recommended to receive a large number of vaccinations—in the United States, infants ≤12 months of age are recommended to receive at least 22 vaccine doses that cover 10 different infectious etiologies (20)—during a time when their microbiotas and immune systems are undergoing dramatic changes (21, 22). To better understand how the early-life microbiome impacts vaccine responses in a highly relevant preclinical vaccine immunogenicity model, we analyzed the longitudinal fecal microbiome of nursery-reared infant rhesus macaques throughout the first eight months of life. These animals were included in previously described pediatric HIV vaccine studies that utilized an HIV gp120 envelope protein-based strategy (23–25). Although the field has since moved towards alternative strategies that focus on eliciting broadly neutralizing antibodies (26), these data still enabled us to identify microbial features that are associated with an improved antibody response to vaccination. We first determined the key factors—age, birth year, and vaccination regimen—that impact composition of the early-life gut microbiota. Furthermore, we found that a *Sutterella* species, its metabolic pathways, and the production of short-chain fatty acids (SCFAs) and bile acids (BAs) all positively correlate with vaccine-induced, immunogen-specific antibodies. Our results provide critical insight into how the gut microbiota drives humoral immune responses following vaccination. Moreover, the development of new, more effective vaccines may incorporate these identified microbial features to help improve vaccine responses.

## 2 Methods

### 2.1 Cohort information and sample collection

**Figure 1:**
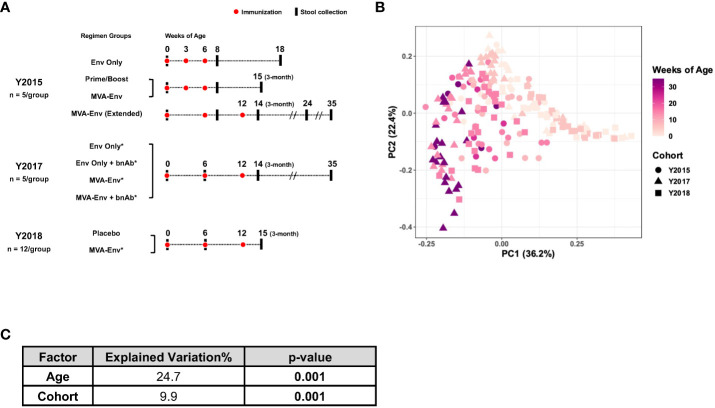
Composition of the gut microbiome in infant macaques is largely driven by age. **(A)** Overview of animal cohorts and stool samples analyzed in this study. Full descriptions of the Y2015, Y2017, and Y2018 cohorts are provided in references (23–25), respectively. Env, HIV gp120 envelope protein; Prime/Boost, a modified vaccinia virus Ankara (MVA) vector expressing HIV Env (MVA-Env) given at birth and boosted with HIV Env; bnAb, broadly neutralizing antibody; *received ChAdOx1.tSIVconsv239 at birth. **(B)** Principal coordinates analysis of weighted UniFrac distances between gut microbial communities of all samples included in the study. **(C)** Marginal PERMANOVA results for data in panel B to determine the contribution of age and cohort to the observed variability.

We collected a total of 215 stool samples from 64 vaginally-delivered, nursery-reared infant rhesus macaques (*Macaca mulatta*) raised at the California National Primate Research Center from 2015 to 2018. Note that rhesus macaques are seasonal breeders with births occurring in the spring to early summer each year. The fecal material from infant macaques were frozen at -80 °C until further use. These animals received different HIV immunization regimens, as previously described (23–25). In brief, vaccination strategies tested by these studies contained an HIV gp120 envelope (HIV C.1086 Env) protein that was administered with or without the HIV Env protein expressed by the modified vaccinia virus Ankara vector (MVA-Env). For all Y2017 animals and the MVA-Env group in Y2018, a chimpanzee adenovirus vector (ChAdOx1.tSIVconsv239) expressing SIV Gag/Pol was also given at birth. For the Y2017 cohort, the regimen groups included animals that received Env protein only, the Env protein with a dose of broadly neutralizing antibodies (bnAbs) at birth, MVA-Env, and MVA-Env with a dose of bnAbs at birth (see **Figure 1A**). No other regimen group in the Y2015 or Y2018 cohort received bnAbs. During this 4-year study period, infant animals were primarily fed a milk-based formula supplemented with iron (Enfamil); however, some other formulas and fluids were used at times. Specific information regarding the formula received by each individual animal is not available. Samples were collected from animals in each cohort at the times indicated in **Figure 1A**, with a few samples that were unable to be collected. These missing samples include the 0 weeks of age timepoint for 2 animals from Y2015 (1 from MVA-Env and 1 from Prime/Boost) and 4 animals from Y2017 (2 from MVA-Env + bnAb, 1 from Env Only, and 1 from Env Only + bnAb) and the 3 months of age timepoint for 1 animal from Y2015 (MVA-Env, extended).

### 2.2 16S rRNA gene sequencing of fecal samples

DNA was extracted from the fecal samples using a PowerSoilPro DNA Kit (Qiagen) according to the manufacturer’s instructions. The V4 region of the 16S rRNA gene was PCR-amplified (primers 515F and 806R, which carried unique barcodes that allow for multiplexed sequencing), as previously described (27). Amplicons were quantified using a Qubit dsDNA HS assay kit (ThermoFisher), pooled in equimolar concentration to facilitate the analysis of individual samples, and sequenced on a MiSeq sequencer (Illumina).

### 2.3 Bioinformatic analysis of 16S rRNA gene sequencing data

Demultiplexed, adapter-free 16S rRNA gene sequencing data generated from fecal samples were imported into R (version 4.2.2) and processed by the standard pipeline of the DADA2 package (28): paired-end reads were quality filtered, trimmed, denoised, and merged into an amplicon sequence variant (ASV) table followed by chimera removal and taxonomy assignment based on the SILVA 132 database (29). A phylogenetic tree was constructed *de novo* for all ASVs by the DECIPHER and phangorn packages in R (30, 31). Normalization of the ASV table by relative abundance, computation of weighted and unweighted UniFrac distances from the phylogenetic tree, and principal coordinates analysis (PCoA) embedding these distances were executed by the phyloseq R package (32). DESeq2 was run with default thresholds and standard procedures to detect differentially enriched taxa at the ASV level (33), filtering for those that were present in ≥25% of the animals in either group. PICRUSt2 was used to impute metagenomic information from the ASV table (34). To identify MetaCyc pathways that were significantly contributed by an ASV of interest, we focused on pathways that had a relative abundance that positively correlated (*P*<0.05) with the relative abundance of the ASV.

### 2.4 Metabolomic analysis of fecal samples

Fecal samples were analyzed using a custom short-chain fatty acids (SCFA) assay to quantify 12 SCFAs (35) and a bile acids (BA) kit (Biocrates) to quantify 20 BAs. Data collection was performed using UPLC-MS/MS on a Waters Xevo TQ mass spectrometer. Data analysis, which included integration, calibration, and concentration calculations, was done using the Skyline software (www.skyline.ms) for SCFAs and the Waters application TargetLynx™ for BAs. Concentrations were initially reported in μM and converted to nmol/g using the mass of fecal matter delivered. Values below the lower limit of quantification (LLOQ) for SCFAs or the lower limit of detection (LOD) for BAs were replaced by the LLOQ or LOD indicated for each analyte. Four SCFAs and three BAs with concentrations below the LLOQ or LOD in more than 40% of samples were disregarded from further analysis.

### 2.5 Measurement of HIV Env-specific antibodies

HIV gp120-specific plasma IgG was assessed by enzyme-linked immunosorbent assay, and HIV gp120-specific salivary IgA and IgG were determined by binding antibody multiplex assay, as previously described and the data previously reported (23–25). For the ELISAs, microtiter plates were coated with 1086.cΔ7 gp120K160N, blocked, incubated with plasma samples, and bound IgG antibodies were detected with peroxidase-labeled anti-monkey IgG and trimethylbenzene solution. For the binding antibody multiplex assay, 1086.cΔ7 gp-120-conjugated fluorescent magnetic beads were used. Prior to performing IgA assays, specimens were depleted of IgG using protein G Sepharose (GE Healthcare).

### 2.6 Statistical analysis

We performed permutational multivariate analysis of variance (PERMANOVA) using the adonis function from the vegan R package to test the effect of age, cohort, and vaccine regimen on composition of the microbiota (36). Pearson’s correlations were determined between the relative abundance of ASVs, the relative abundance of metabolic pathways, the levels of short-chain fatty acids and bile acids, and/or concentrations of HIV gp120-specific antibodies, with p-values adjusted by the Benjamini–Hochberg procedure to correct for multiple comparisons (per antibody) using the cor.test and p.adjust functions in R (version 4.2.2).

## 3 Results

### 3.1 Composition of the gut microbiome in infant macaques is largely driven by age

We obtained 215 longitudinal fecal samples from 64 vaginally-delivered, nursery-reared infant rhesus macaques raised at a single animal facility. These animals were part of three previously described HIV vaccine studies that, for convenience, we refer to by the animals’ birth years (“Y2015”, “Y2017”, and “Y2018”), which was unique between studies (**Figure 1A**) (23–25). We analyzed the 16S rRNA gene sequences from these samples to longitudinally profile the gut microbiome of nursery-reared infant macaques through the first 35 weeks of life, during which time they were vaccinated with different HIV vaccine regimens. Principal coordinates analysis (PCoA) that embedded the weighted UniFrac distances between the gut microbial communities in all samples revealed an age-dependent maturation of the fecal microbiota from 0 to 35 weeks of age irrespective of cohort (PERMANOVA *P*=0.001; **Figures 1B, C**), a finding consistent with earlier macaque and human studies that demonstrated rapid changes in the microbiota during the early stages of life (22, 37, 38). While age explained ~25% of the variability in the data, the year of birth (i.e., cohort) contributed ~10% of the total variation in the dataset (PERMANOVA *P*=0.001; **Figure 1C**). This cohort effect could be related to either different birth years, with all the environmental differences specific to that year, and/or the different vaccine regimens used in the various studies.

### 3.2 Infants born in different years at the same facility harbor distinct microbiota

**Figure 2:**
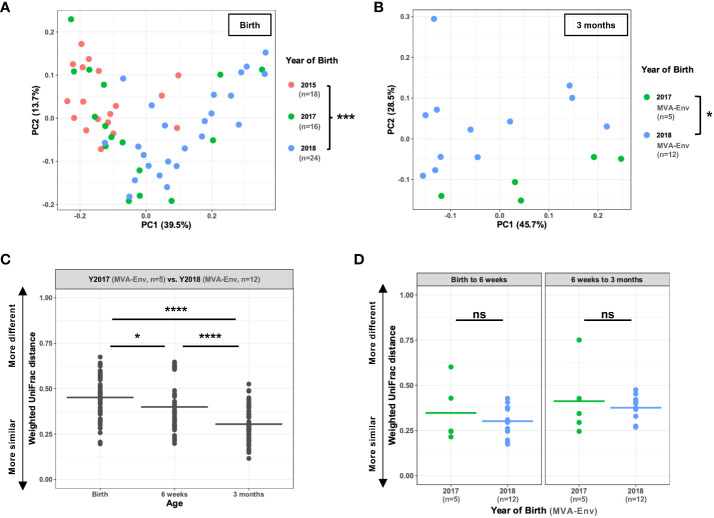
Infants born in different years at the same facility harbor distinct microbiota. **(A, B)** Principal coordinates analysis of weighted UniFrac distances between gut microbial communities of infants born in different years measured at birth **(A)** and, for Y2017 (*n*=5) and Y2018 (*n*=12) animals receiving the same MVA-Env vaccine regimen, at 3 months of age **(B)**. **(C)** Weighted UniFrac distances when comparing animals in Y2017 (*n*=5) and Y2018 (*n*=12) at the indicated ages. All animals received the same MVA-Env vaccine regimen. Note that this analysis compares each of the Y2017 animals to each of the Y2018 animals, which results in a total of 60 comparisons per time point. **(D)** For animals born in 2017 (*n*=5) or 2018 (*n*=12) that received the same MVA-Env vaccine regimen, the weighted UniFrac distances are shown when comparing the same animal at birth and 6 weeks of age (left panel) or 6 weeks and 3 months of age (right panel). ns, not significant; *P<0.05; ***P=0.001; ****P<0.0001 by PERMANOVA [factor = birth year; **(A, B)**], one-way ANOVA with *post-hoc* Tukey’s HSD test **(C)**, or unpaired t-tests **(D)**.

To begin to disentangle the effects of year of birth from vaccination regimen, we compared samples that were collected soon after birth and before the first immunization (i.e., 0 weeks of age). Intriguingly, we found a drastically different microbial composition in newborn animals that were born in different years (PERMANOVA *P*=0.001; **Figure 2A**). This finding demonstrates a marked shift in the abundant gut microbes present in infant rhesus macaques born and nursery-reared at this animal care facility between the years 2015 and 2018. To determine whether the observed differences persisted as the animals grew older, we compared the fecal microbiome of animals born in different years that received the exact same vaccine regimen (i.e., HIV gp120 envelope protein co-administered with MVA-Env). Consistent with our findings with the week 0 stool samples, we found that birth year-dependent effects on the microbiome continued to be evident at 6 weeks and 3 months of age (PERMANOVA *P*=0.001 and 0.015, respectively; **Figure 2B** and **Supplementary Figure 1**). However, the level of dissimilarity between birth years significantly decreased over time (one-way ANOVA *P*=7.6 x 10^-11^; **Figure 2C**), which indicates that the effect of birth year on the fecal microbiome gradually diminishes as the animals age. Moreover, we found no effect of birth year on the rate of maturation of the microbiome within individual animals, regardless of whether we compared monkeys between 0 and 6 weeks of age or 6 weeks and 3 months of age (**Figure 2D**). Taken together, these results establish that macaques born and reared in the same animal care facility in different years have distinct fecal microbiotas through at least the first three months of life, raising implications for study design in this nonhuman primate (NHP) model.

### 3.3 Vaccination impacts the early-life gut microbiome

**Figure 3:**
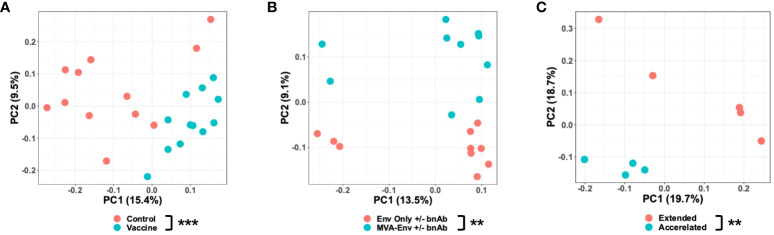
Vaccination impacts the early-life gut microbiome. Principal coordinates analysis of unweighted UniFrac distances between gut microbial communities of 6-week-old Y2018 animals that received a placebo (empty MVA) or vaccine (MVA-Env) at birth **(A)**, 14-week-old Y2017 animals who received either the Env only +/- bnAbs or MVA-Env +/- bnAbs vaccine regimens **(B)**, and 3-month-old Y2015 animals who received the MVA-Env regimen on an accelerated or extended interval **(C)**. **P<0.01; ***P=0.001 by PERMANOVA (factor = vaccine regimen).

Having determined that age and birth year impact the microbiota, we next sought to determine whether early-life vaccination also impacted the microbiota. We first ensured that animals born in the same year but assigned to different vaccine regimens had a similar microbiota prior to immunization by confirming there was no detectable difference in the microbial composition of the week 0 fecal samples (**Supplementary Figures 2A–C**). We then focused on animals from the Y2018 cohort that were vaccinated with either a placebo (an empty MVA vector) or an HIV gp120-based vaccine (MVA-Env). We found that animals receiving the HIV vaccine had a significantly different microbiota than control animals as early as 6 weeks after the first immunization (PERMANOVA *P*=0.001; **Figure 3A**). Interestingly, these differences were only observed using an unweighted UniFrac distance and not with a weighted UniFrac distance (**Supplementary Figure 3**), a finding that indicates vaccination mainly impacts rare members of the microbiota. While this result shows that receipt of a vaccination causes a change in the microbiota, it also suggests that immunogen may matter given that the empty MVA vector given to the placebo group contains some immunogenic proteins. To address this question more specifically, we focused on the Y2017 cohort, in which animals were vaccinated with either just the Env protein or if the regimen also included MVA-Env (see **Figure 1A**). Although these animals also differed in whether they received a dose of broadly-neutralizing antibodies (bnAbs) at birth, we confirmed that the administration of bnAbs did not significantly alter the microbiota in either the MVA-Env or Env Only groups (**Supplementary Figures 4A, B**). As such, we combined groups that differed only in their receipt of bnAbs. Notably, we found the inclusion of MVA-Env in the regimen induced changes in the fecal microbiome (PERMANOVA *P*=0.009; **Figure 3B**). Interestingly, significant differences in the microbiota persisted between infants vaccinated by the MVA-Env and Env Only regimens through 35 weeks of age (PERMANOVA *P*=0.019; data not shown). Furthermore, we compared the microbiome of animals from the Y2015 cohort that received the same MVA-Env vaccine but with different immunization intervals, which revealed that the dosing interval of vaccines also induce remarkable differences in the gut microbial communities (PERMANOVA *P*=0.009; **Figure 3C**). Collectively, our results demonstrate that early-life vaccination, including the specific formulation and immunization schedule, alters the microbiota.

### 3.4 A *Sutterella* species positively correlates with vaccine-elicited HIV gp120-specific antibodies

**Figure 4:**
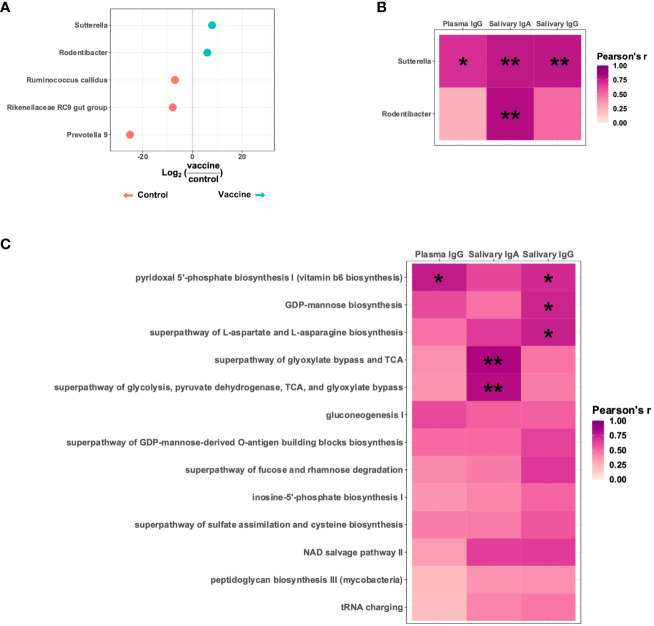
A *Sutterella* species positively correlates with vaccine-elicited HIV gp120-specific antibodies. **(A)** Differentially abundant taxa present in Y2018 animals receiving a placebo (empty MVA) or an HIV vaccine (MVA-Env). **(B)** Pearson’s correlation between the titers of HIV immunogen-specific antibodies and the relative abundance of the two taxa more abundant in vaccinated animals. **(C)** Pearson’s correlation between the titers of HIV immunogen-specific antibodies and the relative abundance of PICRUSt2-inferred metabolic pathways contributed by and significantly correlated with the *Sutterella* species enriched in the vaccinated animals. *P_adj_ <0.05; **P_adj_ <0.01 by Pearson’s correlation adjusted by the Benjamini-Hochberg procedure.

Given that the microbiota is a key regulator of the development and maintenance of the immune system, we reasoned the microbiota may modulate vaccine responses. In an effort to keep age, birth year, and the specifics of the vaccine regimen constant, we focused on the Y2018 cohort given this study had the largest number of animals that received the same vaccination strategy (MVA-Env) and also included a placebo control group (immunized by an empty MVA vector) (25). Knowing that vaccination changes the microbiome, we initially compared the microbiomes of animals that received the placebo or HIV vaccine to focus on bacterial taxa promoted by vaccination. At 15 weeks of age, which was 3 weeks after the final immunization, we identified five taxa that were differentially abundant between the groups, with two (a *Sutterella* and a *Rodentibacter* species) being more abundant in the group that received the HIV vaccine (**Figure 4A**). Interestingly, the relative abundance of these two taxa positively correlated with vaccine-induced immunogen-specific antibody levels (**Figure 4B** and **Supplementary Table 1**). The *Sutterella* species strongly correlated with anti-HIV gp120 plasma IgG (Pearson’s r = 0.69; *P*_adj_ = 0.025), salivary IgA (Pearson’s r = 0.78, *P*_adj_ = 0.006), and salivary IgG (Pearson’s r = 0.78, *P*_adj_ = 0.003), and the *Rodentibacter* species strongly correlated with anti-HIV gp120 salivary IgA (Pearson’s r = 0.81, *P*_adj_ = 0.003).

Since *Sutterella* correlated with all three antibody types analyzed and has previously been associated with vaccine responsiveness in both humans and animal studies (39–42), we focused further on this taxon. To identify specific *Sutterella*-related metabolic pathways associated with vaccine responsiveness, we used PICRUSt2 to infer the functional profile of the microbiota. Of the metabolic pathways contributed by the identified *Sutterella* species, we identified 13 that positively correlated with the abundance of the *Sutterella* species, which suggests this bacterial taxon significantly contributes to these pathways. Of note, five of these pathways significantly correlated with higher levels of the vaccine-induced anti-HIV gp120 antibodies (**Figure 4C** and **Supplementary Table 2**). These pathways included biosynthesis of vitamin B6, GDP-mannose, and aspartate/asparagine as well as two separate but related pathways that reflect energy metabolism.

### 3.5 Fecal short-chain fatty acids and bile acids positively correlate with vaccine-elicited HIV gp120-specific plasma IgG

**Figure 5:**
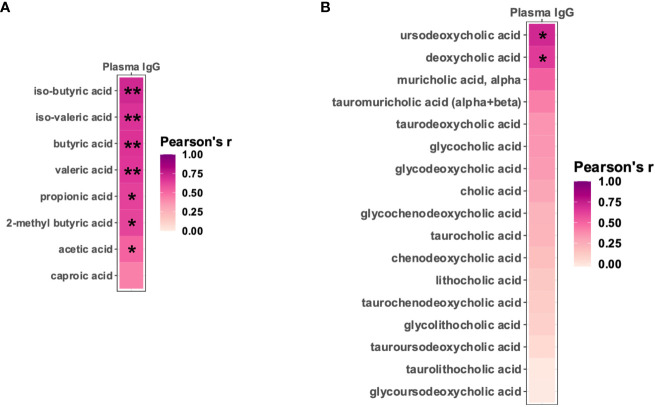
Fecal short-chain fatty acids and bile acids positively correlate with vaccine-elicited HIV gp120-specific plasma IgG. Pearson’s correlation between HIV immunogen-specific plasma IgG and the level of fecal short-chain fatty acids **(A)** and bile acids **(B)**. *P_adj_ <0.05; **P_adj_ <0.01 by Pearson’s correlation adjusted by the Benjamini-Hochberg procedure.

Since many of the *Sutterella* metabolic pathways that positively correlated with the vaccine-induced anti-HIV gp120 antibodies have been linked to the production of short-chain fatty acids (SCFAs) and bile acids (BAs) (43–49), we examined whether fecal levels of these specific metabolites correlated with anti-HIV gp120 plasma IgG in vaccinated infant rhesus macaques. Seven of the eight detectable SCFAs demonstrated significant positive correlations with titers of gp120-specific plasma IgG (**Figure 5A** and **Supplementary Table 3**). In addition, two BAs, ursodeoxycholic acid and deoxycholic acid, strongly correlated with higher levels of anti-HIV gp120 plasma IgG (**Figure 5B** and **Supplementary Table 4**). These results strongly suggest that SCFAs and BAs may represent the functional metabolites through which *Sutterella* modulates vaccine-induced antibody responses.

## 4 Discussion

We longitudinally profiled the early-life gut microbiota in nursery-reared rhesus macaques born at a single animal facility. We found that the fecal microbiota composition in infant rhesus macaques is mostly driven by age, birth year, and vaccination regimens. In addition, we identified a specific *Sutterella* taxon, its metabolic pathways, and the production of SCFAs and BAs that positively correlate with improved antibody responses to vaccination.

Our findings provide valuable insight into the design of future microbiome studies in nonhuman primates (NHPs) by illustrating the key factors that impact the gut microbiota in early life. Although the microbiome is known to vary dramatically during early life (22, 37, 38), we also found that the microbiome of infant macaques differs based on the year of birth, a finding that highlights the importance of controlling for birth year when analyzing the microbiome of nursery-reared infant rhesus macaques. Although these findings are conceptually analogous to studies that have shown significant temporal variations in the gut microbiota of mice obtained from the same vendor (50, 51), this has not previously been demonstrated to be an important issue for NHPs. A potential limitation to our analysis is that the newborn rhesus macaques spent a variable amount of time with their dams before moving to the nursery, which typically occurred within the first 2–3 days of life. In addition, the animals were born to different mothers, and the maternal microbiome is known to impact the microbial compositions of newborns (52). Similarly, different formulas were used at times throughout the 4-year study period, which could also influence the microbiota (53). It is possible these differences, which are common across the three studies—along with other unknown environmental variations that are specific to each birth year—manifest with longer lasting impacts on the microbiome. In this way, we are using “birth year” as a composite variable that incorporates the many environmental differences that exist between these years. Notably, these birth year-dependent differences become smaller as the animals age, which suggests that the effect of birth year is more pronounced at the earliest ages. Although we do not have longitudinal samples throughout infancy and into adulthood to determine whether there is some age at which there is no longer an effect of birth year, the gut microbiota of human infants is known to resemble that of an adult by 2–3 years of age (22, 37, 54). Accordingly, we speculate that this birth year effect may be unique to infant macaques and is not a significant factor in juvenile or adult macaques.

Similar to several other studies (55–60), we found that vaccinations alter the fecal microbiota in rhesus macaques. Interestingly, this effect may be limited to the early-life microbiota of rhesus macaques as infant vaccinations do not induce substantive changes in the microbiota of juveniles (77–88 weeks of age) (61). However, this earlier study of juvenile rhesus macaques focused on a weighted UniFrac distance, which we similarly found was not different in infant rhesus macaques. It is possible that minor constituents of the microbiota are different in juvenile rhesus macaques similar to our observations with infant animals. Furthermore, while most studies have compared vaccination against a placebo control, we also found that both the composition of the vaccine and its dosing regimen impact the microbiota, with the effect of the immunogen on the microbiome lasting at least eight months in infant macaques. Again, these findings highlight the unique aspects of infant populations that require special considerations in study design of vaccine–microbiota relationships. Additional work is needed to more fully address whether infant vaccinations have a long-lasting effect on the gut microbiota.

To complement our finding that infant vaccination alters the microbiota, we discovered specific microbial features that are enhanced by vaccination and positively correlate with vaccine-specific antibody responses. We identified two bacterial taxa enriched in animals that received an HIV gp120 vaccine that are also associated with increased anti-HIV gp120 antibody titers. One of these taxa belongs to the genus *Sutterella*, which has previously been noted to be immunomodulatory, have increased abundance after vaccination, and be predictive of vaccine responsiveness in both humans and animal models (39–42). Our analysis of imputed metagenomic features suggest that *Sutterella* may improve vaccine responses, in part, through the synthesis of vitamin B6 (62), which has been shown to increase vaccine efficacy (63). In addition, our metagenomic analyses suggest SCFAs and BAs may improve vaccine-specific immune responses, a finding consistent with their known roles in modulating the immune system, promoting B cell function, and driving antibody responses (6, 64, 65). Future work should address whether *Sutterella* is causally linked to improved vaccine responses and, if so, the specific mechanisms involved. Since the microbiota has been demonstrated to divert the antibody response to HIV vaccines towards an unproductive target through cross-reactive antibodies (66, 67), it remains to be seen whether *Sutterella* drives antibody responses through a similar mechanism.

In summary, we have characterized, in detail, the early-life gut microbiota in nursery-reared rhesus macaques at a single animal facility throughout the first eight months of life. We found that the major determinants of the microbiota are age, birth year, and infant vaccine regimen. Our study adds to a burgeoning literature demonstrating the impact of vaccination on the microbiota and reveals this relationship is bidirectional in which specific microbial features reciprocally affect vaccine response. We established that a *Sutterella* species is associated with improved vaccine responses, potentially through its production of vitamin B6, SCFAs, and/or BAs. Our study provides new insight into the mechanisms by which the microbiome improves vaccine responses in infants and strengthens the idea that the microbiome serves as an endogenous adjuvant for vaccines, a relationship that can be further harnessed to develop more effective vaccines delivered in early life.

## Data availability statement

The 16S rRNA gene sequencing data presented in the study are deposited in the NCBI Sequence Read Archive repository, accession number PRJNA1027904.

## Ethics statement

The animal study was approved by University of California at Davis Institutional Animal Care and Use Committee. The study was conducted in accordance with the local legislation and institutional requirements.

## Author contributions

DJ: Conceptualization, Data curation, Formal Analysis, Funding acquisition, Investigation, Methodology, Project administration, Software, Validation, Visualization, Writing – original draft, Writing – review & editing. RG: Data curation, Funding acquisition, Investigation, Writing – review & editing. MD: Investigation, Writing – review & editing. HH: Investigation, Writing – review & editing. PK: Investigation, Writing – review & editing. AA: Conceptualization, Resources, Writing – review & editing. KV: Conceptualization, Resources, Writing – review & editing. KD: Conceptualization, Funding acquisition, Resources, Writing – review & editing. SP: Conceptualization, Funding acquisition, Resources, Writing – review & editing. NS: Conceptualization, Formal Analysis, Funding acquisition, Methodology, Project administration, Supervision, Validation, Visualization, Writing – original draft, Writing – review & editing.
