# Supplementary material for: Sutterella and its metabolic pathways positively correlate with vaccine-elicited antibody responses in infant rhesus macaques
Source: Front Immunol. 2023 Dec 6;14:1283343. doi: 10.3389/fimmu.2023.1283343 (PMC10731017; doi:10.3389/fimmu.2023.1283343)
Supplement: Supplementary file 1 [file DataSheet_1.pdf]

Supplementary Material

1 Supplementary Tables

Supplementary Table 1. Pearson's correlation between the titers of vaccine-elicited HIV gp120-specific antibodies and the relative abundance of the two taxa more abundant in vaccinated animals.

Antibody	Taxon	Pearson's r	P	P_{adj}
Plasma IgG	Sutterella	0.692	0.013	0.025
Plasma IgG	Rodentibacter	0.260	0.415	0.415
Salivary IgA	Sutterella	0.776	0.003	0.003
Salivary IgA	Rodentibacter	0.810	0.001	0.003
Salivary IgG	Sutterella	0.779	0.003	0.006
Salivary IgG	Rodentibacter	0.496	0.101	0.101

Supplementary Table 2. Pearson's correlation between the titers of vaccine-elicited HIV gp120-specific antibodies and the relative abundance of PICRUSt2-inferred metabolic pathways contributed by and significantly correlated with the *Sutterella* species enriched in the vaccinated animals.

Antibody	Pathway	Pearson's r	P	P_{adj}
Plasma IgG	superpathway of L-aspartate and L-asparagine biosynthesis	0.477	0.117	0.304
Plasma IgG	superpathway of fucose and rhamnose degradation	0.404	0.193	0.318
Plasma IgG	gluconeogenesis I	0.608	0.036	0.166
Plasma IgG	superpathway of glycolysis, pyruvate dehydrogenase, TCA, and glyoxylate bypass	0.369	0.238	0.318
Plasma IgG	NAD salvage pathway II	0.342	0.276	0.327
Plasma IgG	GDP-mannose biosynthesis	0.602	0.038	0.166
Plasma IgG	inosine-5'-phosphate biosynthesis I	0.364	0.245	0.318
Plasma IgG	peptidoglycan biosynthesis III (mycobacteria)	0.216	0.500	0.542
Plasma IgG	superpathway of GDP-mannose-derived O-antigen building blocks biosynthesis	0.494	0.103	0.304
Plasma IgG	pyridoxal 5'-phosphate biosynthesis I (vitamin b6 biosynthesis)	0.767	0.004	0.047
Plasma IgG	superpathway of sulfate assimilation and cysteine biosynthesis	0.441	0.151	0.318
Plasma IgG	superpathway of glyoxylate bypass and TCA	0.383	0.220	0.318
Plasma IgG	tRNA charging	0.196	0.542	0.542
Salivary IgA	superpathway of L-aspartate and L-asparagine biosynthesis	0.654	0.021	0.083

Salivary IgA	superpathway of fucose and rhamnose degradation	0.447	0.145	0.192
Salivary IgA	gluconeogenesis I	0.531	0.076	0.164
Salivary IgA	superpathway of glycolysis, pyruvate dehydrogenase, TCA, and glyoxylate bypass	0.810	0.001	0.009
Salivary IgA	NAD salvage pathway II	0.638	0.026	0.083
Salivary IgA	GDP-mannose biosynthesis	0.478	0.116	0.189
Salivary IgA	inosine-5'-phosphate biosynthesis I	0.417	0.177	0.192
Salivary IgA	peptidoglycan biosynthesis III (mycobacteria)	0.368	0.239	0.239
Salivary IgA	superpathway of GDP-mannose-derived O-antigen building blocks biosynthesis	0.498	0.099	0.185
Salivary IgA	pyridoxal 5'-phosphate biosynthesis I (vitamin b6 biosynthesis)	0.619	0.032	0.083
Salivary IgA	superpathway of sulfate assimilation and cysteine biosynthesis	0.443	0.149	0.192
Salivary IgA	superpathway of glyoxylate bypass and TCA	0.830	0.001	0.009
Salivary IgA	tRNA charging	0.421	0.173	0.192
Salivary IgG	superpathway of L-aspartate and L-asparagine biosynthesis	0.748	0.005	0.036
Salivary IgG	superpathway of fucose and rhamnose degradation	0.662	0.019	0.059
Salivary IgG	gluconeogenesis I	0.535	0.073	0.119
Salivary IgG	superpathway of glycolysis, pyruvate dehydrogenase, TCA, and glyoxylate bypass	0.442	0.150	0.162
Salivary IgG	NAD salvage pathway II	0.649	0.023	0.059
Salivary IgG	GDP-mannose biosynthesis	0.733	0.007	0.036
Salivary IgG	inosine-5'-phosphate biosynthesis I	0.513	0.088	0.127
Salivary IgG	peptidoglycan biosynthesis III (mycobacteria)	0.384	0.218	0.218
Salivary IgG	superpathway of GDP-mannose-derived O-antigen building blocks biosynthesis	0.631	0.028	0.060
Salivary IgG	pyridoxal 5'-phosphate biosynthesis I (vitamin b6 biosynthesis)	0.720	0.008	0.036
Salivary IgG	superpathway of sulfate assimilation and cysteine biosynthesis	0.561	0.058	0.107
Salivary IgG	superpathway of glyoxylate bypass and TCA	0.466	0.127	0.160
Salivary IgG	tRNA charging	0.457	0.136	0.160

Supplementary Table 3. Pearson’s correlation between vaccine-elicited HIV gp120-specific plasma IgG and the level of fecal short-chain fatty acids.

Antibody	SCFA	Pearson’s r	P	P_{adj}
Plasma IgG	iso-butyric acid	0.719	0.001	0.007
Plasma IgG	iso-valeric acid	0.677	0.003	0.007

Plasma IgG	butyric acid	0.675	0.003	0.007
Plasma IgG	valeric acid	0.663	0.004	0.007
Plasma IgG	propionic acid	0.631	0.007	0.010
Plasma IgG	2-methyl butyric acid	0.621	0.008	0.010
Plasma IgG	acetic acid	0.522	0.032	0.036
Plasma IgG	caproic acid	0.430	0.085	0.085

Supplementary Table 4. Pearson's correlation between vaccine-elicited HIV gp120-specific plasma IgG and the level of fecal bile acids.

Antibody	BA	Pearson's r	P	P_{adj}
Plasma IgG	cholic acid	0.280	0.276	0.586
Plasma IgG	chenodeoxycholic acid	0.170	0.515	0.795
Plasma IgG	deoxycholic acid	0.640	0.006	0.048
Plasma IgG	glycocholic acid	0.343	0.178	0.489
Plasma IgG	glycochenodeoxycholic acid	0.222	0.392	0.696
Plasma IgG	glycodeoxycholic acid	0.326	0.201	0.489
Plasma IgG	glycolithocholic acid	0.092	0.725	0.880
Plasma IgG	glycoursodeoxycholic acid	-0.023	0.931	0.944
Plasma IgG	lithocholic acid	0.126	0.630	0.875
Plasma IgG	muricholic acid, alpha	0.516	0.034	0.193
Plasma IgG	taurocholic acid	0.214	0.409	0.696
Plasma IgG	taurochenodeoxycholic acid	0.112	0.669	0.875
Plasma IgG	taurodeoxycholic acid	0.350	0.169	0.489
Plasma IgG	taurolithocholic acid	-0.018	0.944	0.944
Plasma IgG	tauromuricholic acid (alpha+beta)	0.417	0.096	0.407
Plasma IgG	tauroursodeoxycholic acid	0.047	0.859	0.944
Plasma IgG	ursodeoxycholic acid	0.709	0.001	0.024

2 Supplementary Figures

2.1 Legends

Supplementary Figure 1. Infant macaques that received the same vaccine regimen but were born in different years harbored distinct microbiotas at 6 weeks of age. Principal coordinates analysis of weighted UniFrac distances between gut microbial communities of 6-week-old infants born in different years but received the same MVA-Env vaccine regimen. ****P*=0.001 by PERMANOVA (factor = birth year).

Supplementary Figure 2. Animals that ultimately received different vaccine regimens had similar pre-vaccination microbiotas. Principal coordinates analysis of unweighted UniFrac distances

between the week 0 gut microbial communities of infant macaques in the Y2015 (**A**), Y2017 (**B**), and Y2018 (**C**) cohorts. Env, HIV gp120 envelope protein; Prime/Boost, a modified vaccinia virus Ankara vector expressing HIV Env (MVA-Env) given at birth and boosted with HIV Env; bnAb, broadly neutralizing antibody; control: empty MVA placebo; vaccine: MVA-Env. ns, not significant by PERMANOVA (factor = vaccine regimen).

Supplementary Figure 3. The abundant members of the microbiota were not altered by vaccination. Principal coordinates analysis of weighted UniFrac distances between the gut microbial communities of infant macaques that received a placebo (empty MVA) or an HIV vaccine (MVA-Env) (Y2018 cohort) at 6 weeks of age. ns, not significant by PERMANOVA (factor = vaccine regimen).

Supplementary Figure 4. Composition of the microbiota was not altered by bnAb. Principal coordinates analysis of unweighted UniFrac distances between the gut microbial communities of 14-week-old macaques in the Y2017 cohort that received the MVA-Env regimen with or without administration of bnAbs (**A**) or the Env only regimen with or without administration of bnAbs (**B**). ns, not significant by PERMANOVA (factor = vaccine regimen)**.**

2.2 Figures

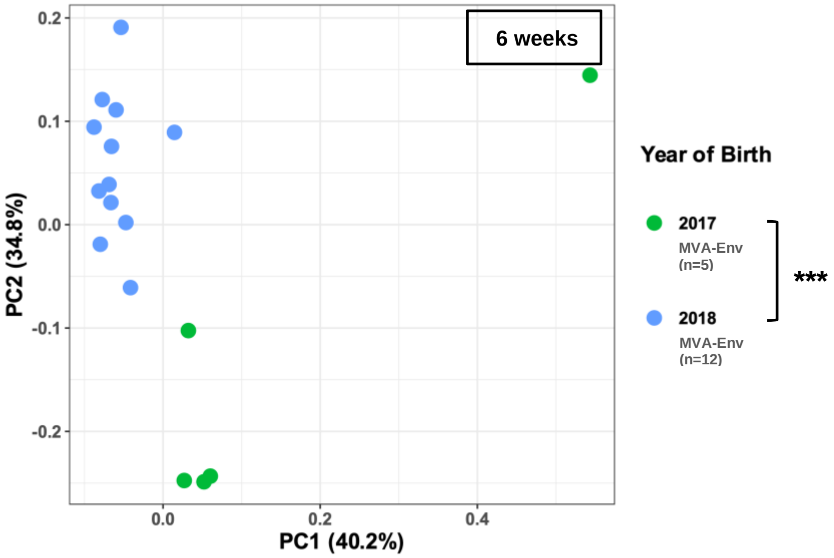

Supplementary Figure 1

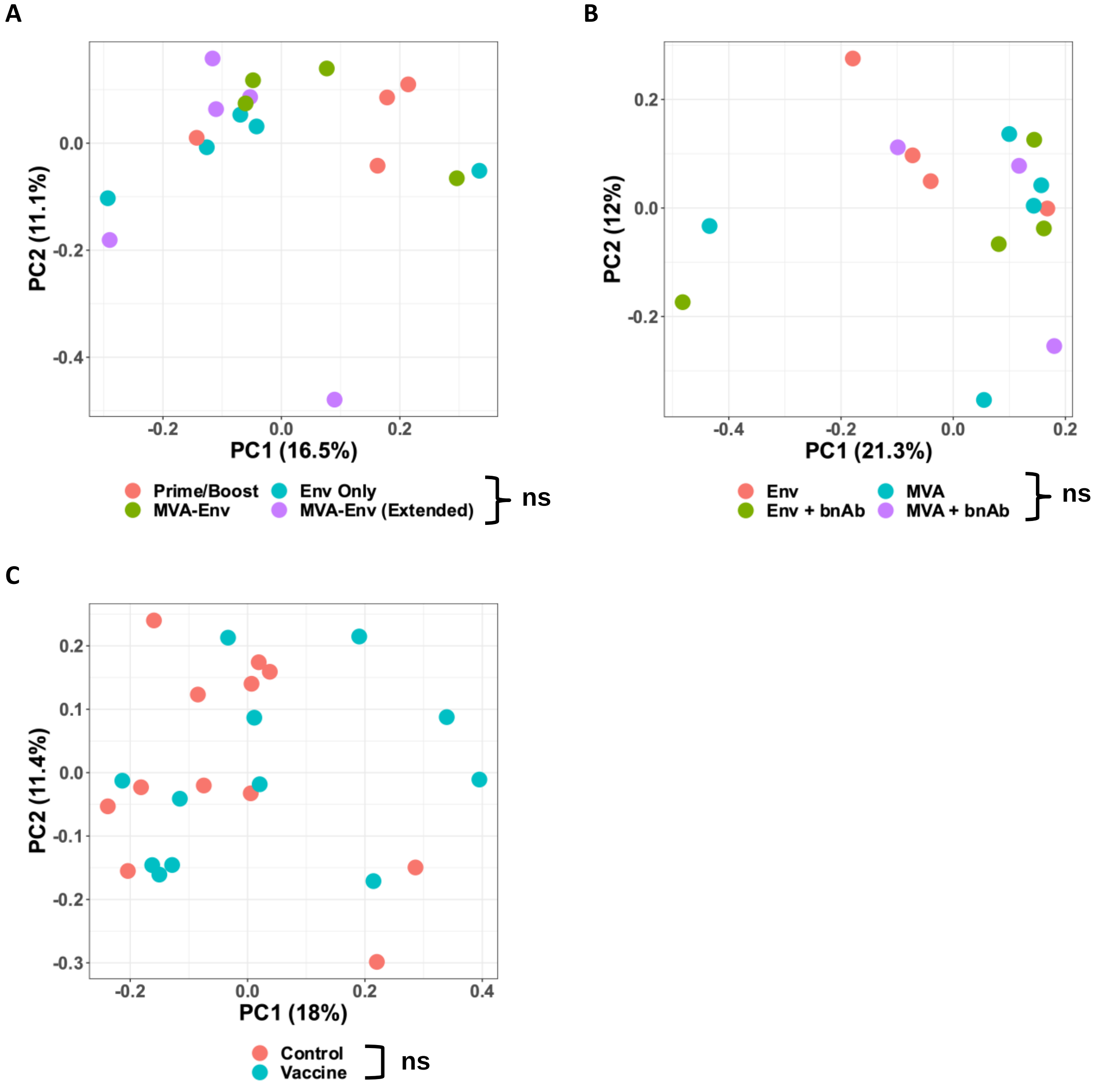

Supplementary Figure 2

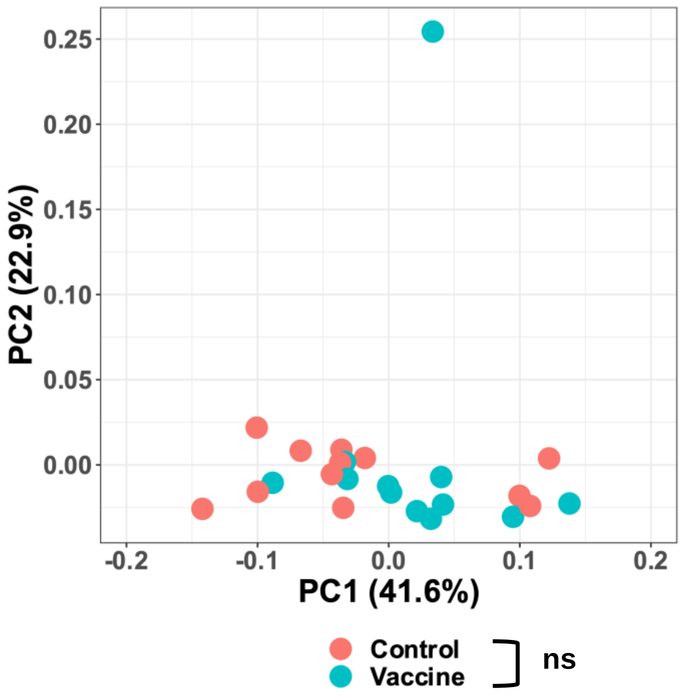

Supplementary Figure 3

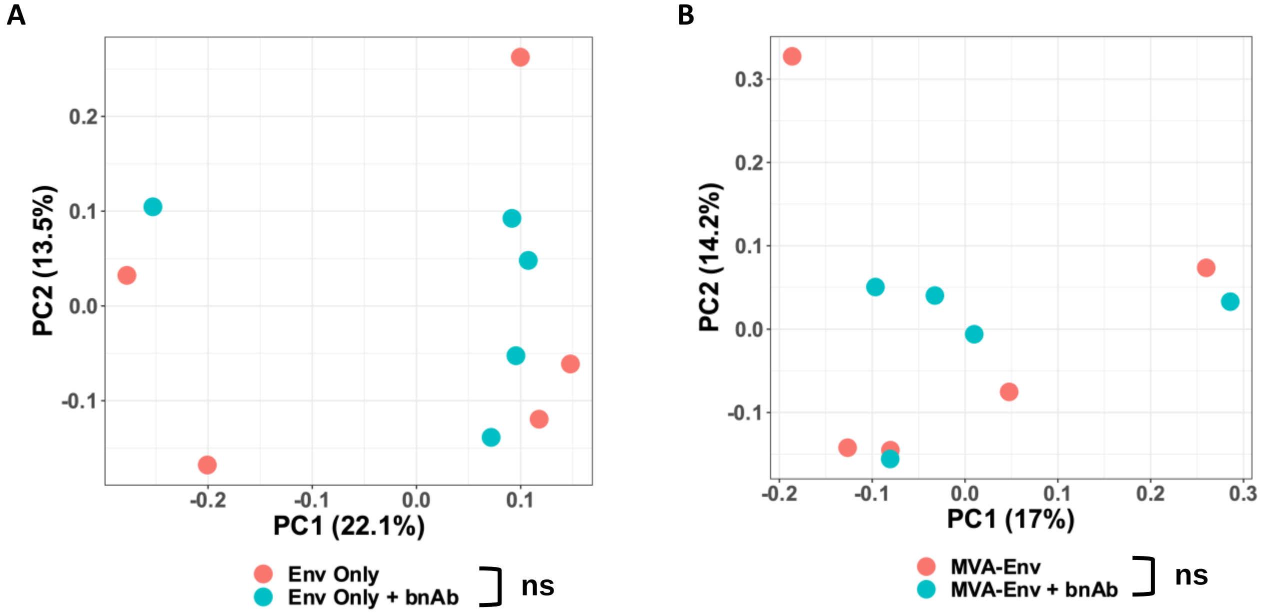

Supplementary Figure 4
